# Diphtheria

**Published:** 1861-04

**Authors:** 


**DIPHTHERIA** is a Greek word signifying skin. Diphtherite, as the French call it, or Diphtheritis, means an inflammation of the skin, as the word "itis" at the end of the name of any part of the body, signifies inflammation, "flaming" of the part. But we have an outside skin and an inside skin, which latter is only a continuation of the former, and covers the internal portion of the body as the true skin covers the outside; this in ternal skin is called the mucous membrane. Hence inflammation of the mucous membrane of the eyelids, of the nose, of the bowels, or of the lungs, is as much diphtheria as the inflammation of the throat or windpipe; but in common speech, it is confined to a peculiar affection of the throat. A thin substance sometimes exudes from trees and hardens on the bark. Diph. theria is an exudation from the inner skin of the throat, the mucous membrane; this appears in patches, which spread, and harden, and thicken, until the windpipe is perfectly closed, and death is inevitable; closes as does the spout of a tea-kettle in limestone countries, by continual accretions. In croup, a less solid substance forms, a kind of phlegm, which is more or less tough, but not solid and compact; it also closes the windpipe completely sometimes, and death ensues; but it is not so leathery in its nature, and is not so difficult of removal. Diphtheria is the more dangerous also, because of the great debility which seizes the patient, and the tendency to destructive ulceration of the parts, a kind of rotting or mortification. The thing then which requires the most instant attention, is the softening of this exuding hardening substance; and next, the prevention of continued exudation; doing something to dissolve and bring away the hardening exudation, and then to close the pores or little mouths of vessels which supply the fluid.

The most efficient and unexceptionable method of softening, and dissolving, and loosening this hardening and dangerous exudation, is that devised by Dr. L. A. Sayre, a distinguished surgeon and physician of New-York City, who puts the patient in a small, close room, makes a flat-iron white hot, suspends it over a pail, pours water on it just fast enough to have every particle evaporated, and before it is cold enough to allow a drop of water to fall into the pail, it is replaced by another hot iron, thus keeping the room full of steam at a temperature of eighty degrees Fahrenheit, for several hours; meanwhile the membrane softens, becomes more liquid, and is cast off; but all this time the patient's strength must be kept up by the most nourishing yet the mildest articles of food, as beef-tea, soup, jellies, ice-cream, etc., allowing bits of ice to melt in the mouth as long as agreeable. Meanwhile, the interior of the fauces, throat, larynx, etc., as far down as can be reached, should be painted with a camel's hair pencil, or soft mop dipped in a solution of twenty to forty grains of nitrate of silver, dissolved in one ounce, that is, two tablespoons of pure water; repeat this painting as often as is necessary to unclog the throat. Where the patient is old enough to use a gargle, employ a tablespoon of powdered alum in a quart of water; Prof. Meredith Reese, of this city, prefers a gargle of two ounces of honey mixed with one ounce each of tincture of capsicum and tincture of myrrh. These are the un-medicinal means to be employed by the family, until a physician arrives, when the case should be placed implicitly in his hands, especially as convalescence is painfully slow and precarious. The terms diphtheria and diphtheritis were introduced by M. Bretonneau, in 1826, to indicate a class of diseases, the distinguishing feature of which was the tendency towards the formation of a false membrane, either on the external or internal skin. He noticed this, says Prof. Reese, of the New-York College and Charity Hospital, as an epidemic in France in 1818, 1825, and 1826. It was observed as an epidemic in 1850 in Haverford West, England. It is clearly a constitutional disease, namely, one in which the whole mass of blood is implicated, caused by a peculiar condition or constituent in the atmosphere; this has led to a general but erroneous impression that "diphtheria is catching." It prevails in families, not because it is communicable under any ordinary circumstances, but because members of the same household breathe a common air. But if that air is made more foul by emanations from diphtheritic patients, those who are well, and who otherwise would have kept well, will have their vitality lowered by breathing this vitiated air, and hence become proportionably liable to disease of any kind, and which would assume this form in preference, just as in any epidemic, most forms of disease run into that which is prevalent. Hence it is best when diphtheria appears in a family, either to keep up a thorough ventilation, or, which is easier, safer, and better, send the children to a place several miles distant.

Diphtheria is essentially a low form of fever, a fever in which the patient rapidly fails in strength, and the whole system is oppressed. Generally it appears in a mild form, now and then it is exceedingly malignant and fatal, and these few latter cases have thrown around the name a terror which shakes the stoutest hearts, just as there are a thousand cases of scarlet fever which recover of themselves, while now and then there occurs one which is suddenly and fearfully fatal.

Croup and scarlet fever and putrid sore throat are uniformly the result of the application of cold, of a cold taken in one of three ways.

First. An only child of sixteen, spent several hours in a dancing-school; the room was warm and she danced a great deal, causing free perspiration over the whole body; at the close, which was about dark of a cold, raw, windy November day, she ran down-stairs and stood on the sidewalk waiting for a companion. She was suddenly chilled, and died in forty-eight hours of malignant, putrid sore throat.

Second. Getting chilled by sitting in a cold, damp room, or at an open window.

Third. Allowing a wet garment to dry on the person, while being still.

The same causes induce diphtheria in a diphtheritic condition of the atmosphere; hence in winter, spring, and autumn, keep little children in-doors the whole of all rainy, thawy, raw, windy days; and of all days, until after breakfast, and from and after one hour before sun-down; give them their supper before dark, and send them to bed as soon as the candles are lighted. Next in importance to prevention, is the premonition of diphtheria, the set of signs which indicate its on-coming, and which are peculiar to itself, premising that when scarlet fever is most prevalent, diphtheria most abounds, as in England in 1858, and in New-York City in 1860, where twice as many persons died of scarlet fever in 1860 as in 1859, and never were so many diphtheritic cases reported here as for 1860.

Sore throat, swelling outside, and an exceedingly offensive breath, are among the very first and most distinctive indications; on opening the mouth, there will be seen on the back part of the throat and tonsils spots of a whitish or grayish white color, with fever and general depression and debility. In the earliest stages, a gargle of salt water should be freely used every fifteen minutes ; a tablespoon of tincture of capsicum to a pint, would be a good addition, as it will be found efficient in rapidly clearing away the accumulations; at the same time, bind flannels around the neck, dipped in salt water, as hot as the patient can bear, renewing every five minutes. The very best advice we can give is simply this, whether diphtheria is in the neighborhood or not, if a child from two to twelve years old complains of a sore throat and has a most offensive breath, send instantly for a physician.

